# Rib Bone Stress Injuries in a Novice Golfer With Chronic Scapular Dysfunction: A Case Report

**DOI:** 10.1155/cro/9535705

**Published:** 2026-03-13

**Authors:** Justin Carrard, George Rublev, Marcelo Bordalo, Leopoldo Buttinoni, Stephen Targett

**Affiliations:** ^1^ Division of Sport and Exercise Medicine, Department of Sport, Exercise and Health, University of Basel, Basel, Switzerland, unibas.ch; ^2^ Department of Sports Medicine, Aspetar Orthopaedic and Sports Medicine Hospital, Doha, Qatar, aspetar.com; ^3^ Sport and Youth Health Unit, Children and Adolescent Surgery, Women’s, Maternal and Child Health Department, Lausanne University Hospital, Lausanne, Switzerland, chuv.ch; ^4^ Sport and Exercise Medicine Centre, Musculoskeletal Medicine Department, Lausanne University Hospital, Lausanne, Switzerland, chuv.ch; ^5^ Institute of Sport Science, University of Lausanne, Lausanne, Switzerland, unil.ch; ^6^ David Tvildiani Medical University, Tbilisi, Georgia; ^7^ Department of Sports Medicine and Orthopedic Surgery, MediClubGeorgia, Tbilisi, Georgia; ^8^ Department of Radiology, Aspetar Orthopaedic and Sports Medicine Hospital, Doha, Qatar, aspetar.com; ^9^ Department of Rehabilitation, Aspetar Orthopaedic and Sports Medicine Hospital, Doha, Qatar, aspetar.com

**Keywords:** bone stress injury, case report, golf-related injury, rib stress injury, scapular dyskinesia

## Abstract

Rib bone stress injuries (BSIs) are rare in athletes but have been reported in rotational sports, such as tennis or golf. Scapular dyskinesia is a well‐established risk factor for shoulder injury, but its contribution to rib BSI has not previously been described. This case concerns a 25‐year‐old man with longstanding left scapular dysfunction, who developed progressive pain in the left medial scapular region 6 months after starting intensive golf training. Clinical examination revealed marked scapular dyskinesia, raising suspicion of muscle atrophy or peripheral nerve involvement. Shoulder and chest MRI excluded neuromuscular abnormalities but demonstrated nondisplaced BSI of the left posterior fifth and sixth ribs. This case report is the first to suggest scapular dyskinesia as a potential biomechanical risk factor for rib BSI in golf. It emphasizes the need to consider underlying scapular dysfunction when evaluating athletes with atypical thoracic pain.

## 1. Introduction

Rib bone stress injuries (BSIs) are relatively uncommon in sport but have been described in golfers [1–4], tennis players [5], and boxers [6]. Most published reports linked these injuries to fatigue of the serratus anterior. However, the potential contribution of chronic scapular dyskinesia as a predisposing factor has not been previously addressed. This case report presents a recreational golfer with longstanding scapular dyskinesia who developed two rib BSI, suggesting a possible contribution of maladaptive scapular biomechanics to rib BSI in rotational sports.

## 2. Case Presentation

### 2.1. History

A 25‐year‐old right‐handed man presented with 2 weeks of left medial periscapular pain. The pain began after a golf session during which he attempted to increase the strength of his swing. There was no history of direct trauma. Despite the pain, he managed to finish the game. Notably, he reported a left shoulder dislocation 15 years earlier, for which he received no rehabilitation, and he has experienced ongoing discomfort since then. The initial injury was treated at another hospital, and his records from that time were not kept.

His sports history shows that he started playing golf 6 months ago and has been practicing four times a week, including both driving range sessions and on‐course play (6–12 holes per session). His left side is the dominant one. He also actively plays bowling three times a week, spending about 3 h at each session. He works in cybersecurity for the military and has no history of illness, regular medication use, or known allergies.

Written informed consent for publication was obtained from the patient. The Aspire Zone Foundation Institutional Review Board determined that formal ethics approval was not required for this single‐patient case report because it involves only one participant and does not meet the definition of human‐subjects research.

### 2.2. Clinical Examination

Physical examination revealed asymmetry of the shoulder girdle. Visible scapular dyskinesia was noted, characterized by protraction and medial winging of the left scapula at rest, as well as marked abnormal scapular motion during shoulder flexion and abduction (see Video S1) Standard clinical tests for scapular dyskinesia were performed, including the scapular assistance test and scapular retraction test, both of which were positive on the left side, consistent with abnormal scapular control.

A focused cervical spine examination revealed a full, pain‐free range of motion and no neurological abnormalities. Cervical spine involvement was therefore considered unlikely.

### 2.3. Radiological Assessment

Initial plain radiography of the left shoulder showed normal alignment and joint spaces, with an osseous fragment adjacent to the inferior third of the glenoid, likely representing an old avulsion injury.

Magnetic resonance imaging (MRI) of the left shoulder revealed a small avulsion of the superior fibers of the subscapularis tendon, with an associated small detached bony fragment and enthesophyte formation at the lesser tuberosity. Notably, there was no fatty infiltration of the shoulder girdle musculature.

**Figure 1:**
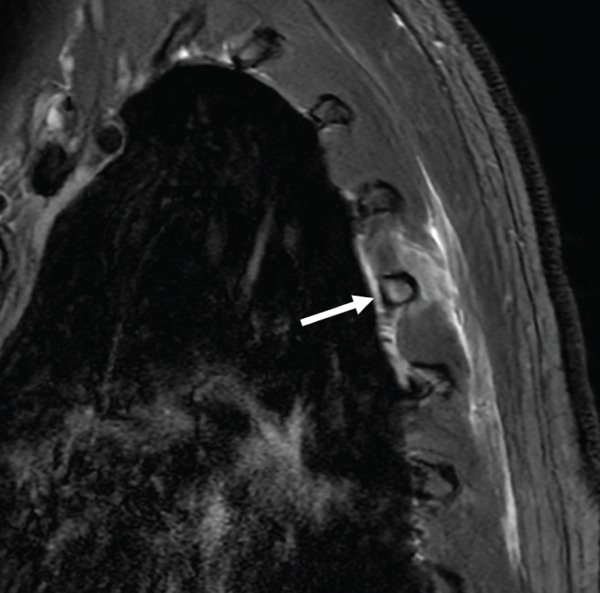
Sagittal T2‐weighted fat‐suppressed MR image of the left thoracic wall. There is bone marrow edema of the fifth left rib (arrow), with surrounding soft tissue edema.

**Figure 2:**
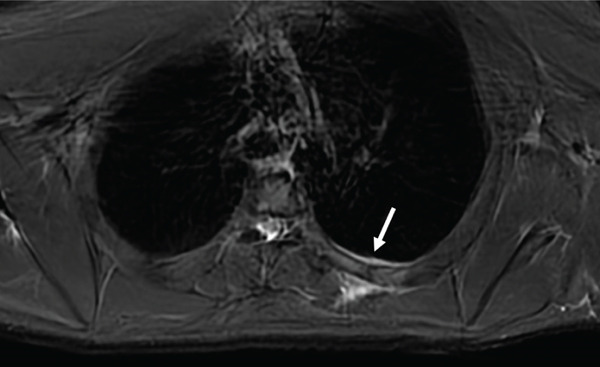
Axial T2‐weighted fat‐suppressed MR image of the thoracic wall. There is bone marrow edema at the posterior aspect of the fifth left rib, with surrounding soft tissue edema. A fracture line is seen (arrow). The finding is consistent with a BSI Fredericson Grade 4b.

To investigate potential neuropathic contributions to the scapular dyskinesis, an MRI of the chest wall with dedicated neurography was performed. Fast spin echo sequences, with and without fat suppression, were acquired in multiple planes. As shown in Figures 1 and 2, there were nondisplaced, incomplete fractures of the posterior segments of the left fifth and sixth ribs, with associated bone marrow edema and soft tissue edema, consistent with a BSI Fredericson Grade 4b. The suprascapular, spinal accessory, and long thoracic nerves appeared normal in caliber and signal intensity. No denervation changes or muscular atrophy were identified in the serratus anterior, trapezius, rhomboids, supraspinatus, or infraspinatus muscles.

### 2.4. Blood Test

Blood test findings were unremarkable (Table 1), supporting a mechanical rather than metabolic origin for the patient’s BSI.

**Table 1:** Laboratory results for the patient, including hematological and biochemical parameters. Reference ranges are provided for each test.

Test	Result	Unit	Reference range
WBC	7.1	10^3^/*μ*L	4–10
RBC	5.61	10^6^/*μ*L	4.5–5.5
Hgb	15.1	g/dL	13–17
Hct	44.9	%	40–50
MCV	80.1	fL	83–101
MCH	26.8	pg	27–32
MCHC	33.5	g/dL	31.5–34.5
RDW‐CV	13.3	%	11.6–14
PLT	254	10^3^/*μ*L	150–410
MPV	7.9	fL	7.5–11.2
Neutrophils	55.0	%	40–80
Lymphocytes	28.7	%	20–40
Monocytes	7.8	%	2–10
Eosinophils	8.2	%	1–6
Basophils	0.3	%	0–2
Iron	12.2	*μ*mol/L	12.5–32.2
TIBC	60.08	*μ*mol/L	44.8–80.6
Iron saturation	20.31	%	15–50
Ferritin	157	*μ*g/L	16–243
LDL	2.62	mmol/L	0–2.59
HDL	1.05	mmol/L	1.03–1.54
Triglycerides	0.91	mmol/L	0–1.69
Cholesterol	4.08	mmol/L	0–5.19
ALT	10	U/L	0–49
AST	14	U/L	0–49
GGT	13	U/L	0–54
Bilirubin total	10.6	*μ*mol/L	5–21
BUN	7.9	mmol/L	2.8–7.2
Creatinine	103.8	*μ*mol/L	59–104
eGFR	88.2	mL/min	> 60
Magnesium	0.79	mmol/L	0.73–1.06
Calcium	2.43	mmol/L	2.2–2.65
Glucose fasting	4.7	mmol/L	4.1–5.6
PTH	43.4	pg/mL	15–68.3
25(OH) vitamin D	35.2	ng/mL	30–80
TSH	2.103	uIU/mL	0.35–4.94
FT3	2.78	pg/mL	1.58–3.91
FT4	1.28	ng/dL	0.7–1.48

*Note:* neutrophils % = percentage of neutrophils in white blood cells; lymphocytes % = percentage of lymphocytes in white blood cells; monocytes % = percentage of monocytes in white blood cells; eosinophils % = percentage of eosinophils in white blood cells; basophils % = percentage of basophils in white blood cells; iron = serum iron; iron saturation = percentage of transferrin saturated with iron; ferritin = serum ferritin; triglycerides = serum triglycerides; cholesterol = total cholesterol; bilirubin total = total serum bilirubin; creatinine = serum creatinine; magnesium = serum magnesium; calcium = serum calcium; glucose fasting = fasting plasma glucose.

Abbreviations: 25(OH) vitamin D, 25‐hydroxy vitamin D; ALT, alanine aminotransferase; AST, aspartate aminotransferase; BUN, blood urea nitrogen; eGFR, estimated glomerular filtration rate; FT3, free triiodothyronine; FT4, free thyroxine; GGT, gamma‐glutamyl transferase; Hgb, hemoglobin; Hct, hematocrit; HDL, high‐density lipoprotein cholesterol; LDL, low‐density lipoprotein cholesterol; MCH, mean corpuscular hemoglobin; MCHC, mean corpuscular hemoglobin concentration; MCV, mean corpuscular volume; MPV, mean platelet volume; PLT, platelet count; PTH, parathyroid hormone; RBC, red blood cell count; RDW‐CV, red cell distribution width–coefficient of variation; TIBC, total iron‐binding capacity; TSH, thyroid‐stimulating hormone; WBC, white blood cell count.

### 2.5. Physiotherapy Assessment

Following the initial medical evaluation, a comprehensive physiotherapy assessment was performed to further characterize scapular mechanics, strength, and functional limitations.

A visible medial apex of the scapula over the upper trapezius indicated scapular winging. Pain was localized to the posterior left medial rib cage. Shoulder flexion was symmetrical at 180° bilaterally. However, total rotation range was reduced on the left, as were external rotation (left: 90°, right: 110°) and seated thoracic rotation (left: 60°, right: 70°); both movements provoked pain on the left. Isometric strength testing revealed pronounced weakness of both internal and external rotation at 90° abduction and a lower IR/ER strength ratio on the left. Thoracic flexion and sit‐up screening elicited moderate discomfort (3/10). Functional golf‐specific movements demonstrated persistent scapular dyskinesis and reduced thoracic mobility, though with gradual improvement in symptoms. Finally, the initial Western Ontario Shoulder Instability Index Summary was 813/2100, and the Quick Disabilities of the Arm, Shoulder and Hand score was 47.7/100.

### 2.6. Diagnosis

A diagnosis of posterior rib BSI Fredericson 4b was made, with chronic scapular dyskinesia considered a possible contributing factor. The latter was attributed to the absence of rehabilitation treatment following the prior shoulder dislocation. The pain likely resulted from the rib BSI due to ineffective load transfer and compensatory movement patterns during repeated golf swings.

The differential diagnosis for medial periscapular and posterior thoracic pain included muscular strain (rhomboids, trapezius, or serratus anterior), intercostal muscle injury, thoracic radiculopathy, peripheral nerve palsy (long thoracic, spinal accessory, or suprascapular nerve), stress reaction without fracture, and metabolic bone disease. Neuropathic causes were excluded by normal chest MRI neurography and the absence of muscle denervation or atrophy. Metabolic and systemic causes were considered unlikely given normal laboratory findings.

### 2.7. Management

Following diagnosis, the patient was managed conservatively with temporary cessation of aggravating activities, particularly golf and bowling. A targeted physiotherapy program focusing on scapular motor control, rotator cuff strengthening, and thoracic mobility was initiated.

## 3. Discussion

This case suggests that chronic scapular dyskinesia may increase susceptibility to rib BSIs when individuals take up new, repetitive upper body activities such as golf. Maladaptive scapular mechanics can alter force transmission, increasing mechanical load and stress concentration across the posterior ribs during trunk rotation and forceful swings.

In addition to golf, the patient regularly played bowling, which may have contributed to cumulative rotational loading of the thorax. Although bowling was performed with the right (dominant) arm, repetitive trunk rotation and deceleration forces may still impose contralateral mechanical stress on the posterior rib cage. While golf was considered the primary precipitating factor due to recent load escalation, the potential additive effect of bowling cannot be excluded.

Rib BSIs in golfers are most commonly reported on the lead side, often attributed to repetitive ground strikes and fatigue of the lead‐side serratus anterior muscle, which is believed to shift mechanical stress to the posterolateral ribs [7]. This case suggests that chronic scapular dysfunction should be considered a potential predisposing factor for rib BSI, particularly in sports that involve repetitive trunk and upper limb movements. A comprehensive biomechanical assessment is key for identifying modifiable risk factors and preventing recurrence. Interdisciplinary collaboration among sports physicians, radiologists, and physiotherapists facilitates accurate diagnosis.

## Author Contributions

Justin Carrard and George Rublev shared first authorship.

## Funding

Open access publishing facilitated by Universitat Basel, as part of the Wiley ‐ Universitat Basel agreement via the Consortium Of Swiss Academic Libraries.

## Disclosure

All content was subsequently reviewed and edited by the authors, who take full responsibility for the final version of the publication.

## Consent

The patient allowed personal data processing, and written informed consent was obtained.

## Conflicts of Interest

The authors declare no conflicts of interest.

## Supporting information


**Supporting Information** Additional supporting information can be found online in the Supporting Information section. Video S1. Clinical examination of the patient who sustained bone stress injuries of the left posterior fifth and sixth ribs 6 months after commencing intensive golf training. The video demonstrates active shoulder range of motion, with abnormal scapular kinematics evident on the left side during arm elevation and lowering.

## Data Availability

The data that supports the findings of this study are available in the Supporting Information of this article.
